# Validity of a simple spillover correction for positron emission tomography measurements in the cerebrospinal fluid region

**DOI:** 10.1007/s12194-025-00972-5

**Published:** 2025-10-02

**Authors:** Emi Hayashi, Shin Hibino, Mitsuhito Mase

**Affiliations:** 1 Planning and Research Bureau, Nagoya City Rehabilitation Agency, 1-2, Mikan-yama, Yotomi-cho, Mizuho-ku, Nagoya, Aichi Japan; 2 Department of Medical Technology, Nagoya City University Rehabilitation Hospital, 1-2, Mikan-yama, Yatomi-cho, Mizuho-ku, Nagoya, Aichi Japan; 3 General Affairs Department, Nagoya City Rehabilitation Agency, 1-2, Mikan-yama, Yatomi-cho, Mizuho-ku, Nagoya, Aichi Japan; 4 Department of Neurosurgery, Nagoya City University Graduate School of Medical Sciences, 1 Kawasumi, Mizuho-cho, Mizuho-ku, Nagoya, Aichi Japan

**Keywords:** Spillover correction, PET, CSF, Partial volume correction, [^15^O]H_2_O

## Abstract

Positron emission tomography (PET) measurements in the cerebrospinal fluid (CSF) region may be overestimated because of spillover artifacts from surrounding radioactivity. In this study, we proposed a simple spillover correction method (subtraction method) and evaluated its validity. A cylindrical phantom simulating brain ventricles was used to compare the subtraction method with the geometric transfer matrix (GTM) correction approach. And the subtraction method was applied to dynamic PET images of [^18^F]fluorodeoxyglucose (FDG), [^18^F]fluorodopa (FDOPA), and [^11^C]raclopride (RAC), and [^15^O]H_2_O (H_2_O). The effects of spillover correction on CSF measurements were assessed. Both methods effectively reduced spillover artifacts in the phantom study. In dynamic PET images, after spillover correction, time–activity curves for FDG, FDOPA, and RAC approached near-zero levels in the CSF, whereas H_2_O continued to show increasing activity over time. This approach effectively reduces artifacts and offers the advantages of simpler volume-of-interest settings and straightforward calculation procedures.

## Introduction

The glymphatic hypothesis [1] regarding the production and absorption of cerebrospinal fluid (CSF) proposes that CSF in the subarachnoid space flows from the perivascular space into the interstitial fluid in the brain, and then flows back into the subarachnoid space via the perivenular space, facilitating the transport of waste materials within the brain parenchyma. Thus, CSF and extracellular fluid play an extremely important role in intracranial material turnover. Water molecules are the major components of CSF and interstitial fluid, and analyzing their dynamics is extremely important for understanding the pathophysiology of intracranial diseases. Quantitative positron emission tomography (PET) imaging is a promising approach for assessing CSF dynamics, but accurate evaluation of the CSF region remains challenging. When measuring radioactivity in PET images, care must be taken to avoid overestimation (spill-in) and underestimation (spill-out) because of partial volume effects (PVE) [2]. To minimize these effects, the measurement area should be at least three times larger than the spatial resolution [3]. However, in actual clinical PET examinations, small regions are often evaluated. CSF region generally shows low radioactivity, whereas the surrounding regions exhibit higher intensity. Therefore, overestimation due to spill-in, referred to as the spillover artifact here, must be considered when measuring CSF radioactivity. To date, no reports have specifically addressed PVE correction methods focused on measuring the CSF region.

In this study, we propose a simple correction method to remove spillover artifacts, which are overestimation components, but do not consider underestimation caused by spill-out from the CFS region. Using a cylindrical phantom simulating the ventricles, we compared the geometric transfer matrix (GTM) method, a typical partial volume effect correction method, with the subtraction method. In addition, the validity of the method will be examined by adapting dynamic PET data from multiple radioactive tracers.

## Materials and methods

### Phantom test

#### Phantom structure and volume of interest (VOI) setting

**Fig. 1:**
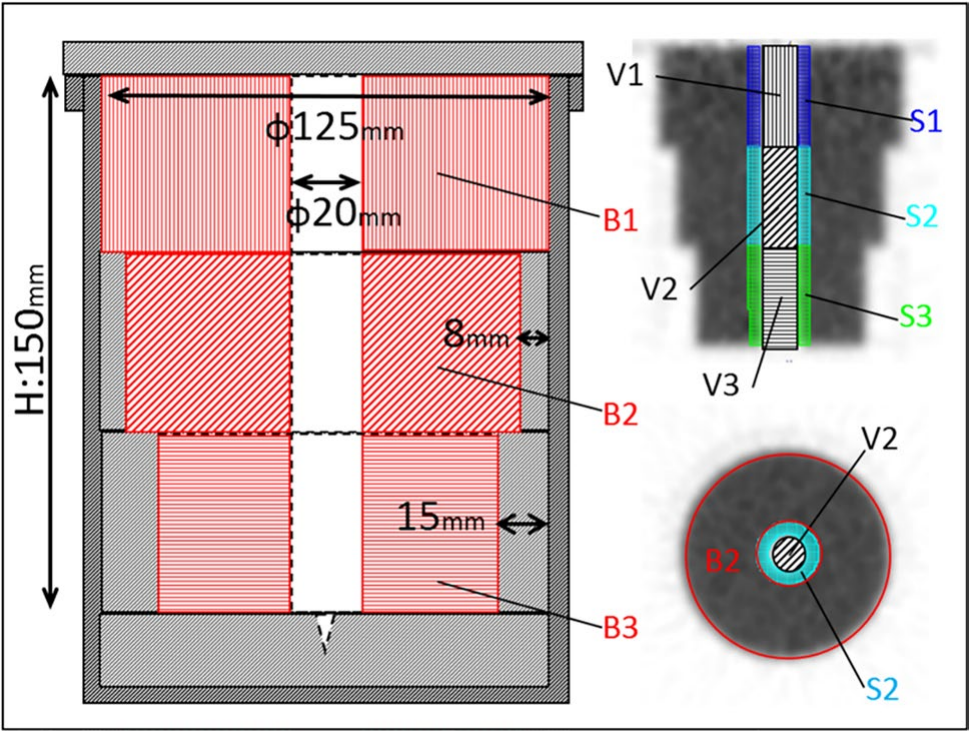
Structure of the cylindrical phantom simulating brain ventricles. The structural schematic of the phantom (coronal section) is shown on the left, and representative PET images are shown on the right. Brain regions are labeled as B1, B2, and B3; ventricular regions as V1, V2, and V3; and surrounding regions of the ventricles as S1, S2, and S3. The brain regions are structures with outer diameters differing by 50 mm intervals

A cylindrical structure simulating the ventricles was placed at the center of the phantom to represent the ventricle (V) region. The phantom was filled with [^18^F] fluorodeoxyglucose (FDG) solution to simulate brain parenchyma and defined as the brain (B) region. The B region consisted of three segments with different outer diameters spaced 50 mm apart, assuming a pattern of different brain sizes (Fig. 1). This phantom was designed in-house and manufactured by Kinjo Lite Co., Ltd. (Nagoya, Japan). Based on the computed tomography (CT) images, VOIs were created for *B*1, *B*2, *B*3, *V*1, *V*2, and *V*3 corresponding to the three outer diameters, and radioactivity was measured. The radioactivity measurements were obtained from PET images at the same location as *b*1, *b*2, *b*3, *v*1, *v*2, and *v*3.

#### PET acquisition and image reconstruction

PET acquisition was performed using a Biograph mCT (Siemens Healthineers, Erlangen, Germany) at three radioactivity levels: 14.6, 29.3, and 58.3 MBq. PET data were acquired in list mode for 300 s, 321 s, and 347 s. The range of the condition of the included radioactivity was based on the peak value of the radioactivity in the cerebral gray matter of a human [^15^O]H_2_O (H_2_O) PET scan. The peak value was approximately 35 kBq/ml (in-house data), and the corresponding dose in the phantom was approximately 50 MBq (volume of the B region was 1386 ml).

Images were reconstructed using filtered back projection with time-of-flight and a Gaussian smoothing filter (full width at half maximum: 4 mm). The matrix size was 400, pixel size 0.51 mm, and slice thickness 2.03 mm. PET images were corrected for normalization, dead time, attenuation, and scatter.

**Fig. 2:**
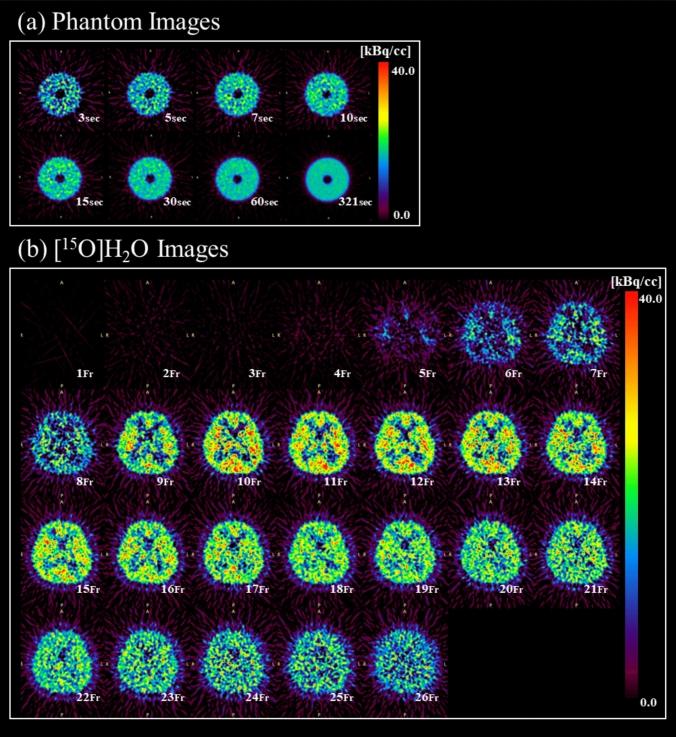
Examples of phantom images under low-radioactivity conditions and 15-min dynamic [^15^O]H_2_O-PET Images. The upper panel **a** shows low-radioactivity phantom images, with durations of 3, 5, 7, 10, 15, 30, and 60 s, and shows 321 s images. The lower panel **b** shows an example of a 15-min dynamic [^15^O]H_2_O-PET image

**Fig. 3:**
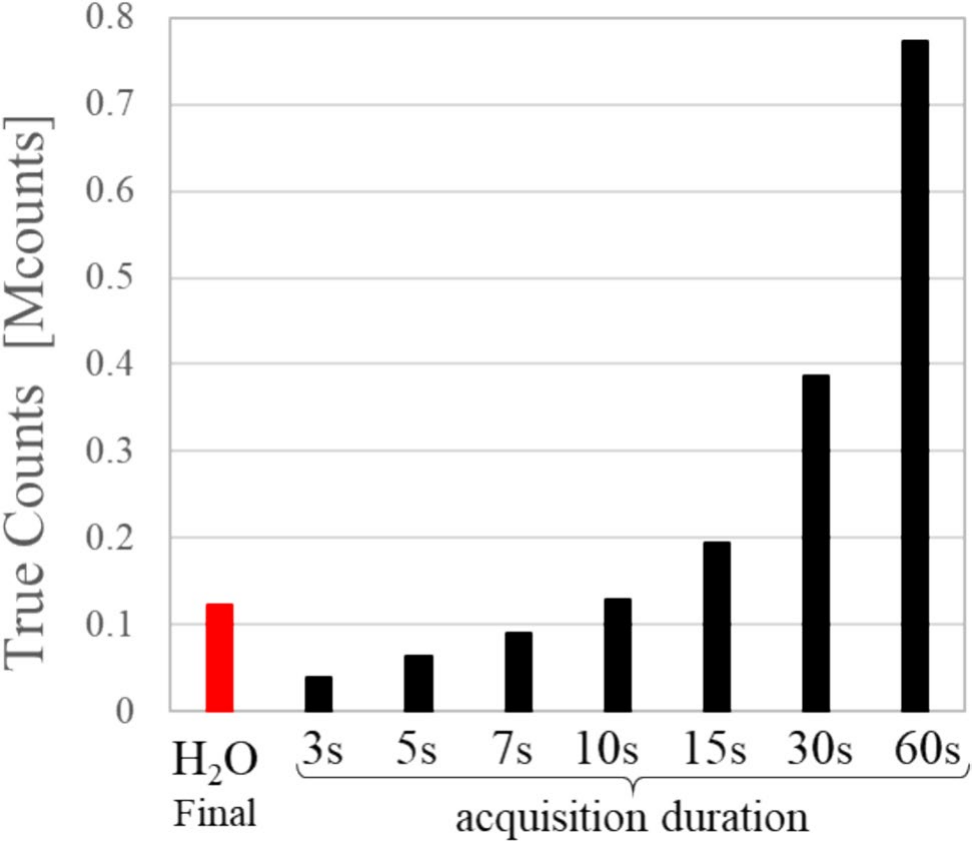
The true count for each image reconstruction condition. The true count for each phantom image at each acquisition duration and for the final frame image of [^15^O]H_2_O-PET was calculated from the PET count rate statistics

Next, image reconstruction was performed for list mode acquisition data of 29.3 MBq level, assuming sufficiently decayed low-radioactivity conditions. Images were created with durations of 3, 5, 7, 10, 15, 30, and 60 s. Considering the effects of statistical variation, three sets of images were created with delay times of 0 s, 60 s, and 120 s. Figure 2a and b shows examples of phantom images and 15-min dynamic H_2_O PET images. The true count of the phantom images obtained for each acquisition duration was calculated from the PET count rate statistics. For reference, Fig. 3 shows the true count under the final frame image conditions of the H_2_O PET data.

#### Resolution calculation

The spatial resolution of the PET image (*R* [mm]) was calculated as 6 mm, using the intrinsic resolution of the Biograph mCT scanner (*R*_1_ = 4.4 mm) and the smoothing filter (*R*_2_ = 4 mm), based on Eq. 1 [4]:

1 $$R=\sqrt{{{R}_{1}}^{2}+{{R}_{2}}^{2}}$$

#### PVE correction using the GTM method

Using PMOD3.5 (PMOD Technologies, Zurich, Switzerland), PVE correction was performed with the VOI-based GTM method described by Rousset et al. [5] to obtain the corrected radioactivity (*GTM_v*) in the V region.

#### Spillover correction using the subtraction method

The image acquired at 14.6 MBq was used as the reference to calculate the spillover correction factor (*F*), and the 29.3 and 58.3 MBq images were used for validation. A 6-mm band surrounding the V region was defined as the surrounding (S) region, representing the spillover source in the V region (Fig. 1). S1, S2, and S3 were created corresponding to the size of B region, and the radioactivity of *s*1, *s*2, and *s*3 was measured. The correction factor *F* was calculated as the ratio of radioactivity in the V region (*v*_ref_) to that in the S region (*s*_ref_) in the reference image (Eq. 2). The corrected value in the V region (*cor.v*) was obtained using Eq. 3. *F* obtained from the reference image was *F*1 = 0.221, *F*2 = 0.202, and *F*3 = 0.216. Spillover correction was performed on the validation image. Average and standard deviation of *cor.v1*, *cor.v2*, and *cor.v3* were calculated to evaluate the phantom test.

2 $$F={v}_{\text{ref}}/{s}_{\text{ref}}$$

3 $$cor.v=v-F\times s$$

#### Numerical analysis

The correlation coefficients between *b*, *v*, and *s* and the included radioactivity were obtained. Average radioactivity and standard deviation were calculated from *b*, *v*, *s*, *GTM_v*, and *cor.v* at three different points for each size of B region.

Next, we evaluated the measured values for *b*,* s*,* v*,* GTM_v*, and *cor.v* under low-radioactivity conditions. We created a graph with acquisition duration on the horizontal axis and measured values on the vertical axis. We performed a one-sample *t *test for *GTM_v* and *cor.v* against the ideal value of radioactivity of 0 [kBq/ml], with a significance level of 0.05.

### Application of the subtraction method in brain imaging of healthy subjects

#### Participant information and ethical approval

Data were obtained from an anonymized image database of healthy individuals previously collected at the Nagoya City Rehabilitation Center. These data were collected based on the following protocols: “Construction of a normal database for examination using a new PET camera (mCT)” (Ethical Review Approval: March 12, 2014). Additionally, image information and PET examination information collected under “Elucidation of the mechanism of spinal fluid production and absorption from the viewpoint of water turnover and its application to the diagnosis of noninvasive hydrocephalus” (Ethical Review Approval: May 27, 2014) were utilized.

Regarding the storage of samples and other materials after the completion of these studies, images and clinical data obtained from the research were stored in the electronic medical record system and image server, with the storage period aligning with the hospital’s internal data retention period for the electronic medical record system and server. The dynamic PET images analyzed included FDG, [^18^F]fluorodopa (FDOPA), and [^11^C]raclopride (RAC) from one 42-year-old man, and H_2_O from one 54-year-old man. Corresponding 3D-T1 and 3D-T2 magnetic resonance imaging (MRI) images were also used. This paper was approved in 2022 as a retrospective observational study using existing data and completed on March 13, 2025. Entire information generated for this study, including images and numerical data produced for analysis, will be permanently deleted five years after the study’s completion.

#### Imaging and reconstruction conditions

PET data collected for 15 min after intravenous administration were used. Images were reconstructed using filtered back projection with time-of-flight and a Gaussian smoothing filter (full width at half maximum: 4 mm). Matrix size was 400, pixel size 1.018 mm, and slice thickness 2.03 mm. PET images were corrected for normalization, dead time, attenuation, and scatter. Image reconstruction followed the same parameters (except for pixel size) as those in the phantom study. For FDG, FDOPA, and RAC, the frame durations were: 5 s × 8 frames, 10 s × 2, 15 s × 4, 20 s × 3, 30 s × 6, and 60 s × 14. For H_2_O, these were: 5 s × 8, 10 s × 2, 15 s × 4, 20 s × 3, 30 s × 4, 60 s × 3, 120 s × 1, and 300 s × 1.

#### Image analysis

**Fig. 4:**
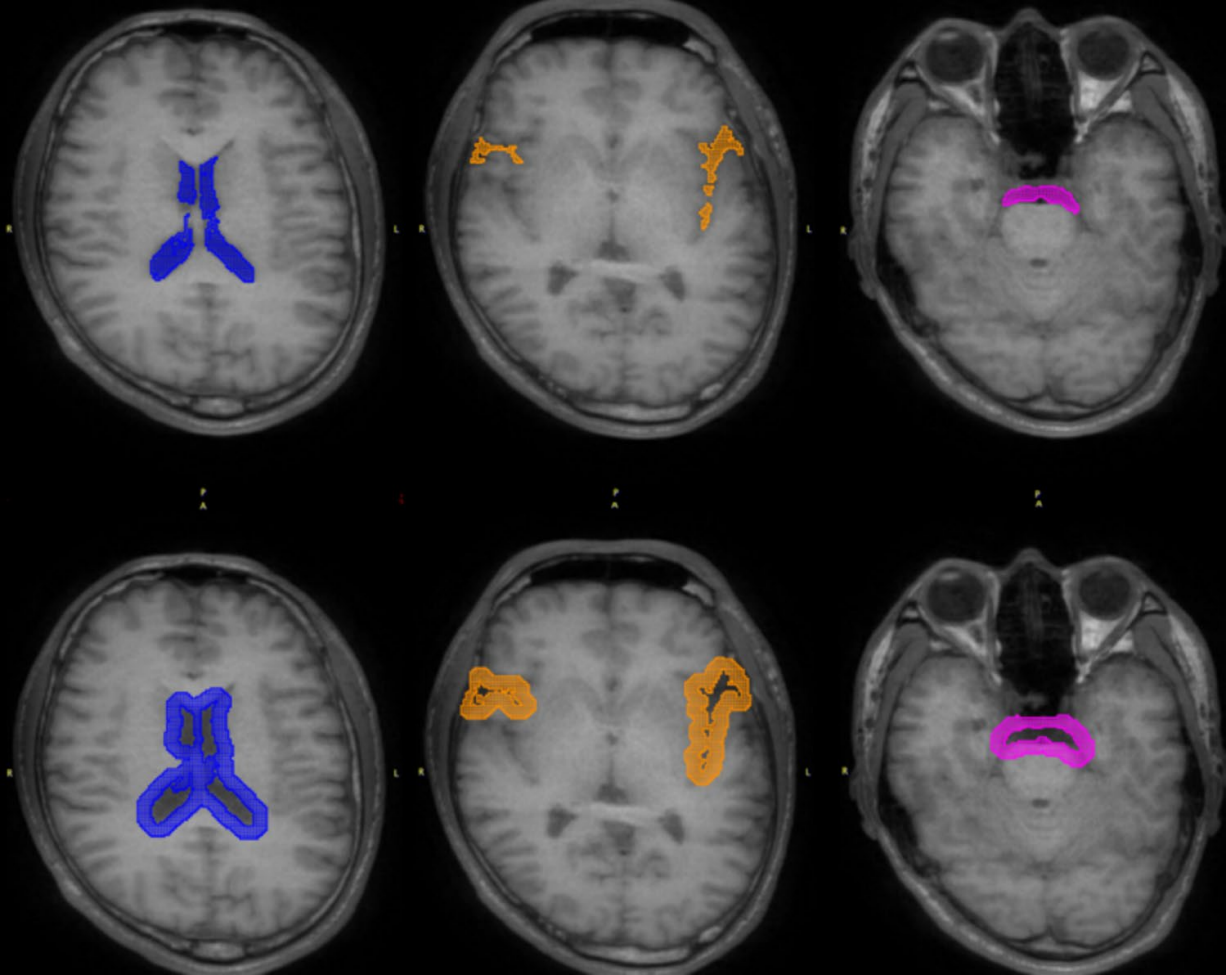
Examples of VOI in the CSF region and surrounding areas. VOIs for the CSF regions (upper row) and their corresponding surrounding regions (lower row) are shown on MRI-T1 images from a healthy individual. The LV is indicated in blue, the FS in orange, and the PPC in pink

PET and MRI images were rigid matched by PMOD3.5 Mutual Information was selected as the dissimilarity function. MRI images (3D-T1) and average PET images of all frames were used. A neurosurgeon manually defined VOIs on the 3D-T1 and 3D-T2 MRI images for three CSF regions, lateral ventricles (LV), Sylvian fissure (FS), and prepontine cistern (PPC). The surrounding region was created by dilation for 6 mm (Fig. 4).

#### Spillover correction using the subtraction method

The time–activity curves (TACs) for the LV, FS, and PPC were labeled as *CSF*(*t*), whereas those of their surrounding regions as *S*(*t*) (Fig. 4). The corrected radioactivity *cor.CSF*(*t*) was calculated using Eq. 4. The correction factor *F* is the point at which *S*(*t*) is large and *CSF*(*t*) is small, i.e., the point at which *CSF*(*t*)/*S*(*t*) is minimum (Eq. 5). The search interval for *F* was 60–900 s, because immediately after administration, the measured values fluctuate significantly due to the influence of high radioactivity in the blood vessels.

4 $$cor.CSF\left(t\right)=CSF\left(t\right)-F\times S(t)$$

5 $$F=CSF(t)/S(t)$$

#### Numerical analysis

TACs for *CSF*(*t*), *S*(*t*), *F* × *S*(*t*), and *cor.CSF*(*t*) were plotted for the LV, FS, and PPC across the four radiotracers. *F *(= *CSF*(*t*)/*S*(*t*)) was plotted on the secondary axis of the same graph. The determined *F* values and their corresponding times are shown.

## Results

### Phantom test

**Fig. 5:**
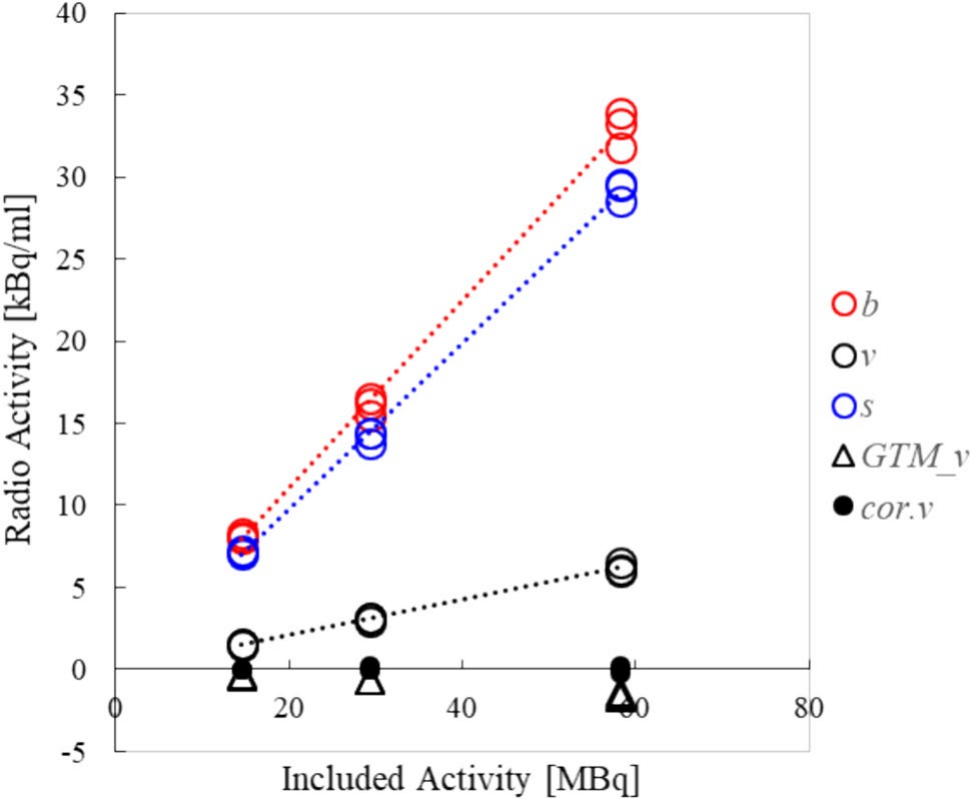
Scatter plot of included radioactivity and measured values. The measured values of *b*, *v*, and *s*, *GTM_v* and *cor.v* are shown. *b* is shown as a red circle, *v* as a black circle, and *s* as a blue circle, and the approximate straight lines are shown as dotted lines. The average radioactivity *b*, *v*, and *s* increased linearly in proportion to the included radioactivity, and the correlation coefficient showed a strong positive correlation of 0.99. The corrected results are shown as triangles for *GTM_v* and filled circles for *cor.v*

Scatter plots of the measured values of *b*, *v*, *s*, *GTM_v*, and *cor.v* against the included radioactivity are shown in Fig. 5. The average radioactivity *b*, *v*, and *s* increased linearly with the included radioactivity, and the correlation coefficient showed a strong positive correlation of 0.99. *GTM_v* and *cor.v* approached zero, effectively correcting the overestimation due to spillover. Table 1 summarizes the results.

**Table 1:** Radioactivity measurements in each VOI from the phantom study

Included activity [MBq]	Average and SD of radioactivity [kBq/mL]				
	*B*	*v*	*s*	*GTM_v*	*cor.v*
14.6	8.12 ± 0.16	1.53 ± 0.05	7.19 ± 0.09	− 0.38 ± 0.08	–
29.32	16.06 ± 0.58	3.1 ± 0.11	14.21 ± 0.33	− 0.65 ± 0	0.08 ± 0.12
58.29	32.99 ± 1.04	6.22 ± 0.29	29.23 ± 0.57	− 1.57 ± 0.14	− 0.01 ± 0.2

Measurements are expressed as the average ± standard deviation (SD) in each volume of interest (VOI) from the phantom study at different included activity levels

*b* values for brain region, *v* values for ventricular region, *s* values for surrounding region, *GTM_v* corrected values using the geometric transfer matrix method, *cor.v* corrected values using the subtraction method

**Fig. 6:**
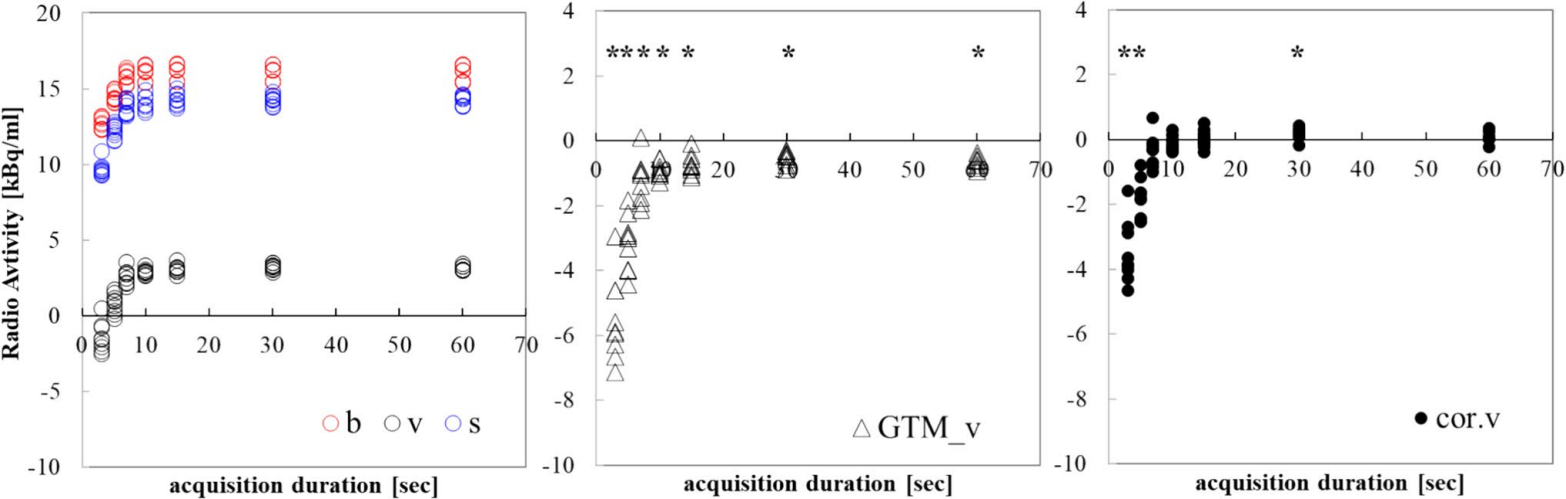
Changes in measured values in low-radioactivity images and results of *GTM_v* and *cor.v*. The left figure shows *b*, *v*, and *s* values from low-radioactivity images reconstructed at each duration; the middle and right figures show *GTM_v* and *cor.v* results, respectively. Asterisks (*) indicate significant differences from 0 [kBq/ml] by a one-sample *t *test (*p* < 0.05)

Figure 6 shows the measured values of *b*, *v*, and *s* and the calculation results of *GTM_v* and *cor.v* in the low-radioactivity image created by changing the acquisition duration. In ultra-low activity images with an acquisition duration of approximately 20 s or less, *b*, *v*, and *s* tended to decrease and variability tended to increase. Affected by the decrease in density, *GTM_v* and *cor.v* also showed similarly low values. A one-sample *t* test was used to compare the corrected results with 0 [kBq/ml] (p < 0.05). *GTM_v* showed significant differences in all images, whereas *cor.v* showed significant differences in the 3-, 5-, and 30-s images.

### Application in brain imaging of healthy subjects

**Fig. 7:**
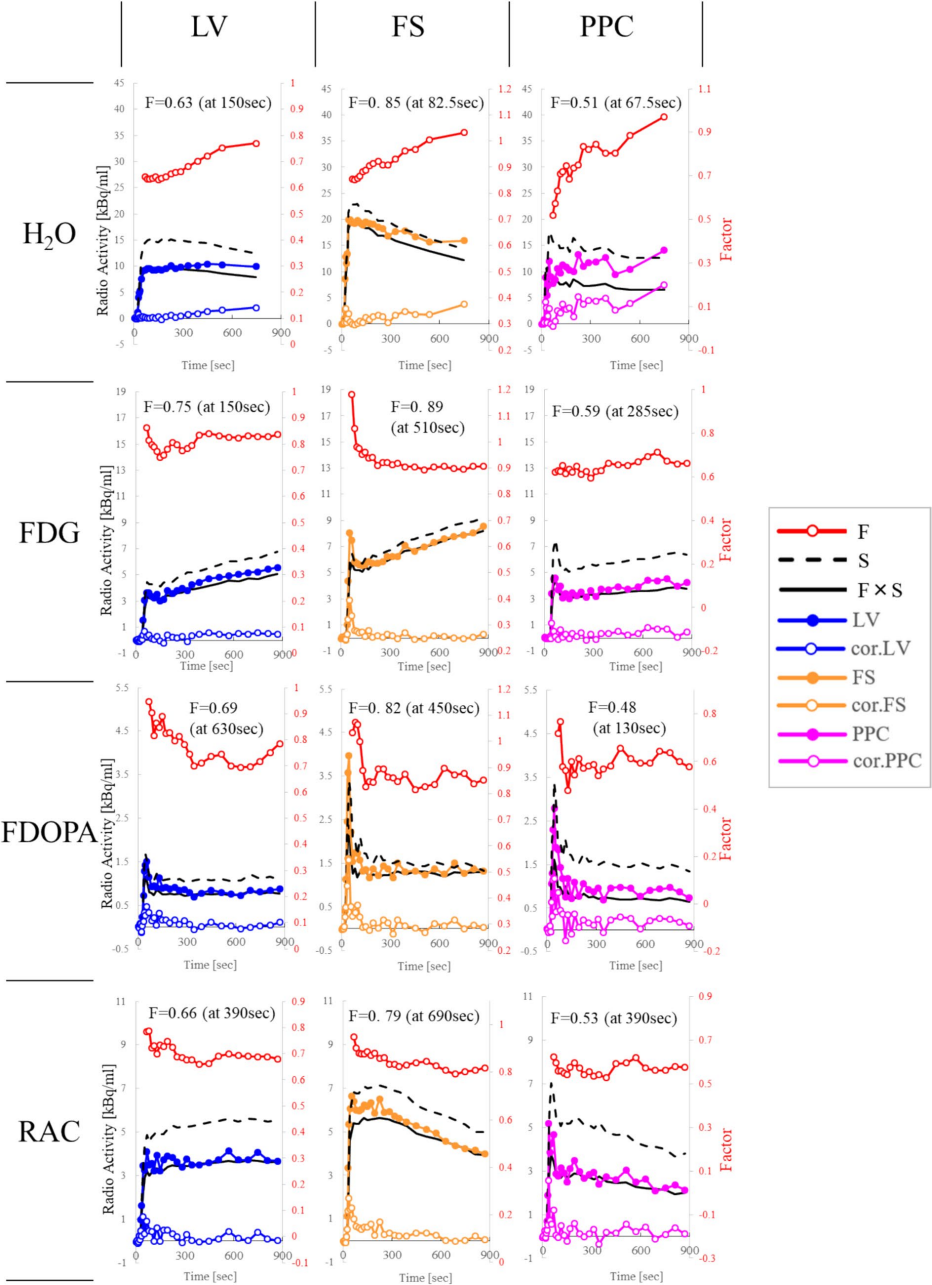
TACs of *CSF*(*t*), *S*(*t*), *F* × *S*(*t*), and *cor.CSF*(*t*) for Healthy Individuals using Four Radiotracers. The left and right columns show results for the LV, FS, and PPC, respectively. The results for H_2_O, FDG, FDOPA, and RAC are shown from the top to bottom of the figure. Closed circles represent *CSF*(*t*), dotted lines represent *S*(*t*), solid lines represent *F* × *S*(*t*), and open circles represent *cor.CSF*(*t*). *F* (= *CSF*(*t*)/*S*(*t*)) was plotted on the secondary axis of the same graph (open red circles with lines). The determined *F* values and their corresponding times are shown

The TACs for *CSF*(*t*), *S*(*t*), *F* × *S*(*t*), and *cor.CSF*(*t*) for the four radiotracers are shown in Fig. 7. LV, FS, and PPC from left to right, and H_2_O, FDG, FDOPA, and RAC results from top to bottom. The closed circle represents *CSF*(*t*), the dotted line represents *S*(*t*), the solid line represents *F* × *S*(*t*), and the open circle represents *cor.CSF*(*t*). *F *(= *CSF*(*t*)/*S*(*t*)) was plotted on the secondary axis of the same graph (open red circles with lines). The determined *F* values and their corresponding times are shown. For all radiotracers, *CSF*(*t*) and *S*(*t*) increased immediately after tracer injection, which then changed gradually with time. FDG, FDOPA, and RAC showed similar trends between *CSF*(*t*) and *S*(*t*), whereas H_2_O demonstrated a slower washout pattern in *CSF*(*t*) compared with *S*(*t*). Consequently, *CSF*(*t*)/*S*(*t*) showed a gradual upward trend for H₂O, while the other three radiotracers remained relatively flat with minor fluctuations. These findings indicate that the artifact component *F* × *S*(*t*) closely approximates *CSF*(*t*) in the cases of FDG, FDOPA, and RAC. After correction, *cor.CSF*(*t*) was near zero for FDG, FDOPA, and RAC but showed a slight increase for H_2_O.

## Discussion

In this study, we propose a simple subtraction method to reduce spillover artifacts in PET measurements of CSF regions, specifically the LV, FS, and PPC. In the phantom study, although the CSF region ideally had zero concentration, measured values were elevated because of spillover from surrounding radioactivity. The linear relationship among B, V, and S regions and the included radioactivity suggested that the artifact component can be estimated using a single coefficient. It was confirmed that the appropriate spillover correction factor *F* was obtained from the reference image and that the overestimation was reduced in the validation image. Both *GTM_v* and *cor.v* were close to zero, reducing the overestimation in the V region. While GTM calculates both spill-out and spill-in, the proposed correction method accounts only for spill-in. Since the V region contained no radioactivity in this phantom experiment, similar results were obtained. Because [^15^O] has a half-life of approximately 2 min, the signal in clinical H_2_O PET images is substantially reduced in the final images acquired at 10–15 min. Under such low-count conditions, image reconstruction leads to a decreased signal-to-noise ratio (SNR), producing noisier images and compromising measurement accuracy, especially when measuring small regions. To simulate low-radioactivity conditions, images were reconstructed using only a portion of the list-mode acquisition data. In ultra-low-count images with short acquisition duration, the measured values in *b*, *v*, and *s* were low (Fig. 6). As reported by Carlier et al. [6], the single scatter simulation with tail fitting and scaling method used in this study can produce inaccurate scatter correction under ultra-low-count conditions. This scatter correction inaccuracy may be leading to underestimation of *GTM_v* and *cor.v*.; however, this cannot explain the present finding, namely the increase in ^15^O-H_2_O during the late phase. A one-sample *t *test against the ideal value of 0 [kBq/ml] showed that *GTM_v* differed significantly at all time points, while *cor.v* differed significantly at 3 s, 5 s, and 30 s. In this experiment, the CSF region was modeled as a 20 mm-diameter cylinder. A detailed investigation using phantoms with complex sizes and shapes remains a future task for comparing the two methods.

Based on the linear relationship observed in the phantom study, we applied the subtraction method to clinical brain PET images. CSF contains relatively large vessels and tissues with abundant blood supply, both inside and around it (for example, the choroid plexus in the ventricles, the middle cerebral artery in the Sylvian fissure, and the basilar artery in the prepontine cistern). In addition, the tissues surrounding the CSF are highly perfused brain regions. As a result, an increase in radioactivity is observed in both *CSF*(*t*) and *S*(*t*) immediately after radiotracer injection. *CSF*(*t*) values increased immediately after radiotracer injection, and for FDG, FDOPA, and RAC, the TACs showed behavior similar to those in the surrounding region *S*(*t*). As these radiotracers are not known to enter or accumulate in the CSF within 15 min, the observed radioactivity in the CSF region was interpreted as resulting primarily from spillover artifacts. In contrast, H_2_O exhibited a slower washout in *CSF*(*t*), and its behavior differed from that of *S*(*t*), indicating a distinct pattern. The relationship between *CSF*(*t*) and *S*(*t*) depends on the tracer. The *F*, defined as *CSF*(*t*)/*S*(*t*), is higher in the first few minutes after injection, reflecting tracer-specific distribution dynamics: H_2_O distributes rapidly due to high permeability, FDG and FDOPA more slowly via carrier-mediated transport, and RAC, being lipophilic, according to blood flow. The radiotracer distribution changed over time; for FDG, DOPA, and RAC, the contrast between *CSF*(*t*) and *S*(*t*) remained relatively constant, suggesting that the spillover correction factor *F* reached a plateau. However, for H_2_O, the correction factor *F* showed a gradual increase, suggesting not only spillover but also actual tracer influx into the CSF. Intravenously administered H_2_O is distributed to brain tissue early in the first pass due to a high extraction fraction of > 90% [7, 8]. The increased *cor.CSF*(*t*) observed in this study indicates that water may diffuse into the CSF compartment during the short imaging window of 15 min. Kudo et al. used MRI to analyze the kinetics of H_2_O with a labeled ^17^O tracer and reported that there is an influx of water into the LV and subarachnoid space immediately after administration [9]. To generalize these results, the included of only one case per tracer is insufficient and represents a limitation of this study. Further validation with a larger number of cases is required.

Accurate delineation of the CSF region is challenging because of the close proximity of blood vessels, nerves, and the choroid plexus. Consequently, applying the GTM method using MRI-T1 images for VOI definition is often difficult. Ibaraki et al. also noted the challenges associated with separating small adjacent tissues with distinct functions when setting VOIs [10]. The subtraction method is a simple correction technique that considers only spillover from the surrounding 6 mm, and although it is not a complete PVE correction method like GTM, it offers practical advantages. Specifically, it avoids the need for complex VOI segmentation and allows for spillover correction using a straightforward calculation, making it useful for clinical applications.

### Limitations

One limitation of this study is that when the image signal is low and the SNR cannot be maintained, measured values may not be reliable. The ROIs were manually defined by a single operator, so further investigation is needed to assess inter-operator variability and reproducibility. Contamination from regions beyond the 6 mm surrounding area used for correction cannot be entirely eliminated, and thus complete correction of overestimation is not achievable. Moreover, because PET resolution differs between the center and periphery of the field of view, correction using a single full width at half maximum has inherent limitations. Due to the finite resolution of PET systems, measurements in small CSF regions are susceptible to contamination, making it difficult to accurately detect tracer influx. Future improvements in PET image reconstruction with PSF-based resolution enhancement, as well as advances in detector technology with higher resolution and sensitivity, are expected to address these challenges.

## Conclusion

The simple subtraction method was able to reduce spillover artifacts in PET measurements in the CSF region, offering a practical approach in situations where conventional GTM-based corrections are difficult to implement.

## Data Availability

The data that support the findings of this study are not openly available due to reasons of sensitivity and are available from the corresponding author upon reasonable request.
